# Hospital Managers and the Insurance Act

**Published:** 1912-06-08

**Authors:** 


### THE EDITOR'S LETTER-BOX.

### Hospital Managers and the Insurance Act.

*To the Editor of* The Hospital.

Sir,—The majority of hospital managers will probably at the present time be considering the position of their employees under the above.

The following point has presented itself to my committee, and I should be glad of an expression of opinion from other hospital managers. In the past the whole of the indoor employees of the majority of hospitals have received medical attendance from the honorary medical staffs, and medicines, and so long as the voluntary character of hospitals continues the same arrangement will prevail.

Is it not possible, therefore, for a case to be presented to the Insurance Commissioners asking for a reduction of contribution payable both by employer and employee in consideration of hospital managers giving a guarantee to provide medical attendance and medicines?—Yours faithfully,

Hospital Secretary.

